# Brain metabolism and cerebrospinal fluid biomarkers profile of non-amnestic mild cognitive impairment in comparison to amnestic mild cognitive impairment and normal older subjects

**DOI:** 10.1186/s13195-015-0143-0

**Published:** 2015-09-15

**Authors:** Artur M N Coutinho, Fábio H G Porto, Fabio L S Duran, Silvana Prando, Carla R Ono, Esther A A F Feitosa, Lívia Spíndola, Maira O. de Oliveira, Patrícia H F do Vale, Helio R. Gomes, Ricardo Nitrini, Sonia M D Brucki, Carlos A. Buchpiguel

**Affiliations:** Department of Radiology/Nuclear Medicine Center/LIM43, Hospital das Clínicas da Faculdade de Medicina da Universidade de São Paulo,Trav. Ovídio Pires de Campos S/N, prédio do Centro de Medicina Nuclear, 2 andar – LIM43, Cerqueira Cesar, São Paulo, CEP 05403-010 Brazil; Department of Neurology...Av. Dr. Enéas de Carvalho Aguiar, São Paulo, 255 CEP 05403-900 Brazil; Department of Psychiatry - R. Dr. Ovídio Pires de Campos, São Paulo, 785 CEP 01060-970 Brazil

## Abstract

**Introduction:**

Mild cognitive impairment (MCI) is classically considered a transitional stage between normal aging and dementia. Non-amnestic MCI (naMCI) patients, however, typically demonstrate cognitive deficits other than memory decline. Furthermore, as a group, naMCI have a lower rate of an eventual dementia diagnosis as compared to amnestic subtypes of MCI (aMCI). Unfortunately, studies investigating biomarker profiles of naMCI are scarce. The study objective was to investigate the regional brain glucose metabolism (rBGM) with [^18^F]FDG-PET and cerebrospinal fluid (CSF) biomarkers in subjects with naMCI as compared to a control group (CG) and aMCI subjects.

**Methods:**

Ninety-five patients were included in three different groups: naMCI (N = 32), aMCI (N = 33) and CG (N = 30). Patients underwent brain MRI and [^18^F]FDG-PET. A subsample (naMCI = 26, aMCI = 28) also had an assessment of amyloid-β, tau, and phosphorylated tau levels in the CSF.

**Results:**

Both MCI groups had lower rBGM in relation to the CG in the precuneus. Subjects with naMCI showed decreased right prefrontal metabolism as well as higher levels of CSF amyloid-β relative to aMCI subjects.

**Conclusion:**

While amnestic MCI subjects showed a biomarker profile classically related to MCI due to Alzheimer’s disease, naMCI patients illustrated a decrease in both prefrontal hypometabolism and higher CSF amyloid-β levels relative to the aMCI group. These biomarker findings indicate that naMCI is probably a heterogeneous group with similar precuneus hypometabolism compared to aMCI, but additional frontal hypometabolism and less amyloid-β deposition in the brain. Clinical follow-up and reappraisal of biomarkers of the naMCI group is needed to determine the outcome and probable etiological diagnosis.

## Introduction

Mild cognitive impairment (MCI) is assumed to be a transitional stage between normal aging and dementia. Clinically, MCI has several etiologies and includes two main subtypes, amnestic MCI (aMCI) and nonamnestic MCI (naMCI) [1]. aMCI forms are typically considered a symptomatic predementia phase of Alzheimer’s disease (AD), while naMCI forms are believed to be more related to other neurodegenerative or nondegenerative conditions, such as vascular or psychiatric disorders [1]. Thus, while the heralding impairment of aMCI is characteristically a memory deficit, the naMCI subtype typically shows a greater preservation of memory function with larger degrees of deficits in the cognitive domains such as attention, language, visuospatial, and executive functions [2, 3]. Previous research has shown that in most cohorts [4, 5], but not all [6], naMCI presented a lower rate of conversion to dementia than the amnestic subtype. Most studies indicate a preferential conversion of aMCI to AD, but other variables such as the presence of more than one affected domain, type of impairment, intensity of cognitive impairment at baseline, and presence of apolipoprotein E epsilon 4 allele may possibly influence the conversion to dementia [7].

According to the most currently accepted theory of AD physiopathology [8], if naMCI is due to disorders other than AD, then biomarkers should be different in aMCI and naMCI. Some authors have compared magnetic resonance imaging (MRI) and positron emission tomography with ^18^F-fluordeoxyglucose ([^18^F]FDG-PET) in both MCI subtypes, concluding that aMCI shows imaging patterns more consistent with a transition to AD, like hypometabolism in the posterior cingulate and temporoparietal regions [8], than naMCI [9–12]. However, most studies have not identified a distinct set of imaging and cerebrospinal fluid (CSF) biomarkers necessary to specifically characterize naMCI, thereby leaving ambiguity in which parameters might best classify nonamnestic forms of MCI.

Given these outstanding issues, the aim of the present study was to compare the profiles of naMCI, aMCI, and control older subjects with [^18^F]FDG-PET (a surrogate marker of synaptic function) and CSF biomarkers (a measurement of brain amyloid deposition and neuronal injury), in efforts to not only illustrate the relationship between naMCI and aMCI CSF biomarkers, but also demonstrate synaptic functioning differences between the groups.

## Materials and methods

### Participants

Community-dwelling older adults (≥60 years old) were recruited through community announcements and invited to participate in meetings hosted in recreation centers throughout the city of São Paulo, Brazil. Subjects reporting any cognitive complaints were evaluated by a neurologist (FHGP). Cognitive complaints needed to be reported by the subjects and were confirmed by a collateral source, usually a relative or spouse. To be included in the study, subjects had to have completed 4 years or more of formal education, or what constitutes “primary education” in Brazil. Older volunteers without cognitive complaints were also recruited as members of the control group (CG). All participants underwent a complete neurological and psychiatric evaluation, a comprehensive neuropsychological test battery, and an assessment for symptoms of depression and anxiety. After each initial assessment, a final diagnosis was established by a consensus of neurologists with expertise in cognitive and behavioral neurology.

The revised Petersen et al. [13] criteria were used to diagnose individuals with MCI. Classification of aMCI or naMCI was determined according to the presence or absence of impairment in memory tasks (which defines aMCI), or other cognitive domains in the neuropsychological assessment [3, 13]. We considered a cognitive function or domain to be impaired if the test *Z* scores on that function were greater than 1.5 below the appropriate mean for age and education on one test, or if the *Z* scores were between 1.0 and 1.5 below the appropriate mean on more than one test of the same cognitive domain [14]. MCI patients were classified into aMCI or naMCI according to the criteria described. The domains impaired in the naMCI group were: executive/attention (*n* = 25), executive/attention and language (*n* = 5), and executive/attention and visuospatial function (*n* = 2).

The cognitive battery used to screen subjects included the Mini-Mental State Examination (MMSE) [15, 16] and the Brief Cognitive Battery [17] followed by the clock drawing test [18]. This battery has been shown to have good accuracy for the diagnoses of early dementia [19]. The following neuropsychological tests were used to define naMCI and aMCI: memory tests (visual reproduction and logical memory subtests of the Wechsler Memory Scale (revised), delayed recall of the Rey–Osterrieth Complex Figure, and the Rey Auditory Verbal Learning Test); constructive abilities (Block Design subtest of the Wechsler Adult Intelligence Scale, and copy of the Rey–Osterrieth Complex Figure); visual perception (matrix reasoning); and attention and executive functions (Trail Making Test (Parts A and B) and the Stroop Test). Application, scoring, and interpretation of the results obtained for all tests were performed according to each reference guide. Scores were adjusted according to appropriate age and education norms. Additional details of the evaluation protocol used, and neuropsychological tests administered, are reported in de Gobbi Porto et al. [20].

Exclusion criteria included: volunteers with clinically relevant psychiatric symptoms meeting DSM-IV criteria (the Geriatric Depression Scale (GDS) [21] and the Geriatric Anxiety Inventory [22] were administered to all MCI patients to evaluate the intensity of depressive and anxiety symptoms, respectively; patients with scores >5 on the GDS were excluded); any uncompensated clinical comorbidity, such as cardiac failure or anemia; history or presence of signs of neurologic diseases, such as Parkinson’s disease, epilepsy, inflammatory disease or stroke, with the exception of migraine; presence of any drug abuse (especially alcoholism); cognitive decline intense enough to interfere with daily activities according to clinical judgment or a score of ≥5 on the Functional Activities Questionnaire [23]; cognitive decline consistent with a diagnostic of dementia according to clinical judgment; diabetes mellitus without adequate glycemic control in the last 2 weeks (excluded because of possible interference with the [^18^F]FDG-PET); and presence of neoplastic or significant vascular lesions on the MRI scan, according to the judgment of a neuroradiologist (EAAFF) and one of the authors (AMNC). Individuals were not excluded based on the presence of white matter hyperintensities (WMH) on the MRI scan. The presence of WMH was an exclusionary criterion only if associated with focal neurological signs or gait impairment in the neurologic examination. No patient was using cholinesterase inhibitors or memantine. Antidepressant use was not strictly exclusionary; patients using antidepressants were allowed to participate if they were on a stable dose for at least 3 months and did not have symptoms of an active psychiatric disease at the time of screening.

This research project was approved by the ethics committee of the Hospital das Clínicas da Faculdade de Medicina da Universidade de São Paulo, and was in agreement with the provisions of the Declaration of Helsinki. All participants signed a term of consent.

### CSF analysis

Of the 65 MCI patients enrolled, 54 (aMCI, 28 patients; naMCI, 26 patients) agreed to have a lumbar puncture to assess amyloid beta peptide (Aβ), tau, and phosphorylated tau (p-tau) protein levels in the CSF. Lumbar punctures were performed after an 8-hour fast, always between 8:00 and 10:00 a.m. A 22-gauge spinal needle was inserted between the fourth or fifth lumbar vertebral body by a trained neurologist. Approximately 10 ml CSF were taken. All of the samples were collected in polypropylene tubes, briefly centrifuged, and stored at a temperature of −80 °C. Total tau, p-tau, and Aβ were determined quantitatively using a commercial sandwich enzyme-linked immunosorbent assay (Innotest hTAUAg Innotest _amyloid 1–42; Innogenetics, Ghent, Belgium).

### MRI acquisition

After the initial work-up, all patients underwent a 3.0 Tesla MRI scan to exclude structural diseases and also for co-registration with [^18^F]FDG-PET images. Following the procedure outlined in Fazekas et al. [24], a neuroradiologist (EAAFF) performed a visual semi-quantitative assessment of a WMH scale, using the axial fluid-attenuated inversion recovery (FLAIR) sequence. The following MRI sequences were acquired: sagittal 3D T1, axial T2 FSE, axial fluid-attenuated inversion recovery (FLAIR), coronal T2 SPIR, and diffusion.

### [^18^F]FDG-PET imaging acquisition

To ensure blood glucose levels lower than 180 mg/ml, participants fasted for at least 4 hours prior to the intravenous injection of 370 MBq [^18^F]FDG in a peripheral vein. Following the tracer injection, patients rested with eyes open and ears unplugged for 60 minutes in a calm, silent and slightly darkened room. Acquisition of the [^18^F]FDG-PET data was run for 15 minutes (matrix = 256 × 256, zoom = 2.5, pixel size = 1.04 mm) using a Siemens Biograph PET-CT scanner (CTI/Siemens, Knoxville, TN, USA). Images were reconstructed with the ordered subset expectation maximization method (with six interactions and 16 subsets) and then smoothed with a 5 mm Gaussian filter. Data were also corrected for scattering, attenuation, and decay. Attenuation correction was performed using single helical computed tomography.

### [^18^F]FDG-PET imaging processing and analysis with SPM8

The following procedures were validated previously [25] and are used as a standard for PET and single photon emission tomography (SPECT) explorative analyses. All PET images of the participants were co-registered with the their MRI images (volumetric T1 sequence) and spatially normalized in SPM8 software (Wellcome Department of Cognitive Neurology, Functional Imaging Laboratory, London, UK) into a standard stereotactic space, based on the SPM8/Montreal Neurologic Institute space, using a 12-parameter linear affine normalization and a further nonlinear iteration algorithm. This was performed using an SPM8 template for [^18^F]FDG-PET. Each of the scans was also individually smoothed with a Gaussian kernel to reduce the impact of misregistration into template space and to improve the signal-to-noise ratio. Images were then smoothed with a 4 mm full-width Gaussian kernel at half-maximum Gaussian filter. To ensure the analysis only included voxels mapping cerebral tissue, a default threshold of 0.8 of the mean uptake inside the brain was selected. Global uptake differences between brain scans were adjusted using the “proportional scaling” statistical parametric mapping (SPM) option. Once adjusted, radioactive counts for each participant were normalized with the average global counts of each group (i.e., the “global means” normalization approach). The relevant peak voxels were identified in terms of coordinates according to Talairach and Tournoux with the help of the Talairach Client software (Research Imaging Institute, University of Texas Health Science Center, San Antonio, TX, USA), and after conversion from the SPM/Montreal Neurological Institute space [26, 27].

### Statistical analysis

Clinical and CSF data analyses were performed using SPSS version 17.0 (SPSS Inc., Chicago, IL, USA). PET data were analyzed on a voxel-by-voxel basis using the SPM8 software (Wellcome Department of Cognitive Neurology) in conjunction with MATLAB R2009a (The Mathworks In., Natick, MA, USA). An analysis of variance (ANOVA) test was used to search for regional brain glucose metabolism (rBGM) differences across the three groups (naMCI, aMCI, and CG).

Post-hoc analyses with nonpaired *t* tests were used to examine differences between each pair of groups. SPM8 maps were generated with *p* <0.001 and the threshold for significance at the voxel level was set at *p* = 0.001 (*Z* score = 3.09) with a minimum extension of 10 voxels in the corresponding cluster. This is a classical threshold for PET and functional MRI studies [25], and has been used previously in studies related to MCI [9, 11, 28]. The initial exploratory analyses with SPM maps generated a *t* statistic for each voxel, thus constituting statistical parametric maps.

To explore the homogeneity of rBGM alterations between groups in particular areas, the higher *Z* scores within each map were identified, and a volumetric region of interest (ROI) in the corresponding cluster of voxels was generated. Subsequently, numeric values representing [^18^F]FDG uptake measures in that cluster for each individual (after the whole normalization process) were obtained with the toolbox MarsBar for SPM [29] under the option “explore design/files and factors”. With this approach, regions of particular interest—voxels in the precuneus—could be better explored.

A directed analysis of ROIs was also completed with the SPM8 software (small volume correction (SVC)). Areas in the temporoparietal association cortex were chosen based on previous reports of regions with typical metabolic impairment in early AD [30, 31]. The following areas were analyzed: posterior cingulate cortex, precuneus, angular gyrus, middle temporal gyrus, and inferior temporal gyrus.

## Results

Ninety-five volunteers were included and classified into one of three groups: aMCI group (aMCI; *n* = 33), naMCI group (naMCI; *n* = 32), or without cognitive impairment group (CG; *n* = 30). Demographic, clinical, and CSF data are presented in Table 1. The CG had more years of formal education than aMCI patients (mean (M) and standard deviation (SD) of 12.9 (4.9) and 9.2 (4.0), respectively; *p* = 0.017). The MMSE was different between the CG and both MCI groups (M (SD) = 29.0 (1.0), 27.6 (1.5), and 27.8 (1.9) for CG, aMCI, and naMCI, respectively; *p* <0.000). No differences were found in age, gender, education, intensity of depressive or anxiety symptoms, presence of hypertension and diabetes, or MMSE scores among the aMCI and naMCI groups. Visual analysis of WMH [24] disclosed no differences across the groups.

**Table 1:** Demographic, neuropsychological, and CSF data for the sample

	CG (*n* = 30)	aMCI (*n* = 33)	naMCI (*n* = 32)	*p* value (two-tailed)
				Multiple comparison
Age (years)^a^	69.5 (6.4)	72.6 (5.5)	69.8 (5.8)	0.065
Gender (female/male)^b^	24/6	20/13	23/9	0.23
Education (years)^a^	12.9 (4.9)	9.2 (4.0)	11.8 (4.3)	0.018
				CG × aMCI
				(*p* = 0.017)
GDS^c^	–	1.5 (1.3)	1 (1.4)	0.113
GAI^c^	–	6.6 (4.3)	5.1 (4.1)	0.181
Hypertension^b^	–	19 (57 %)	15 (46 %)	0.388
Diabetes mellitus^b^	–	7 (21 %)	3 (9 %)	0.186
MMSE^a^	29.0 (1.0)	27.6 (1.5)	27.8 (1.9)	0.000
				CG × aMCI and naMCI
				(*p* <0.003)
CSF Aβ^c,d^	–	704 (248)	918 (430)	0.044
CSF tau^c,d^	–	258 (125)	247 (110)	0.84
CSF p-tau^c,d^	–	44 (12)	43 (14)	0.97
CSF p-tau/Aβ protein^d^		0.07 (0.05)	0.06 (0.04)	0.13
WMH visual scale^b^ [24]	0: 06 (20 %)	0: 04 (12 %)	0: 04 (13 %)	0.525
	I: 13 (43 %)	I: 16 (49 %)	I: 18 (56 %)	
	II: 08 (27 %)	II: 09 (27 %)	II: 10 (31 %)	
	III: 03 (10 %)	III: 04 (12 %)	III: 0 (0 %)	

Data presented as mean (standard deviation)

^a^Analysis of variance (post-hoc test: Bonferroni)

^b^Chi-square test

^c^ *t* test

^d^Subsample of 54 subjects (26 naMCI patients and 28 aMCI patients), Mann–Whitney test (not normally distributed)

*aMCI* amnestic mild cognitive impairment, *Aβ* amyloid beta, *CG* control group, *CSF* cerebrospinal fluid, *GAI* Geriatric Anxiety Scale, *GDS* Geriatric Depression Scale (15 items), *MMSE* Mini-Mental State Examination, *naMCI* nonamnestic mild cognitive impairment, *p*-*tau* phosphorylated-tau, *WMH* white matter hyperintensities

**Fig. 1:**
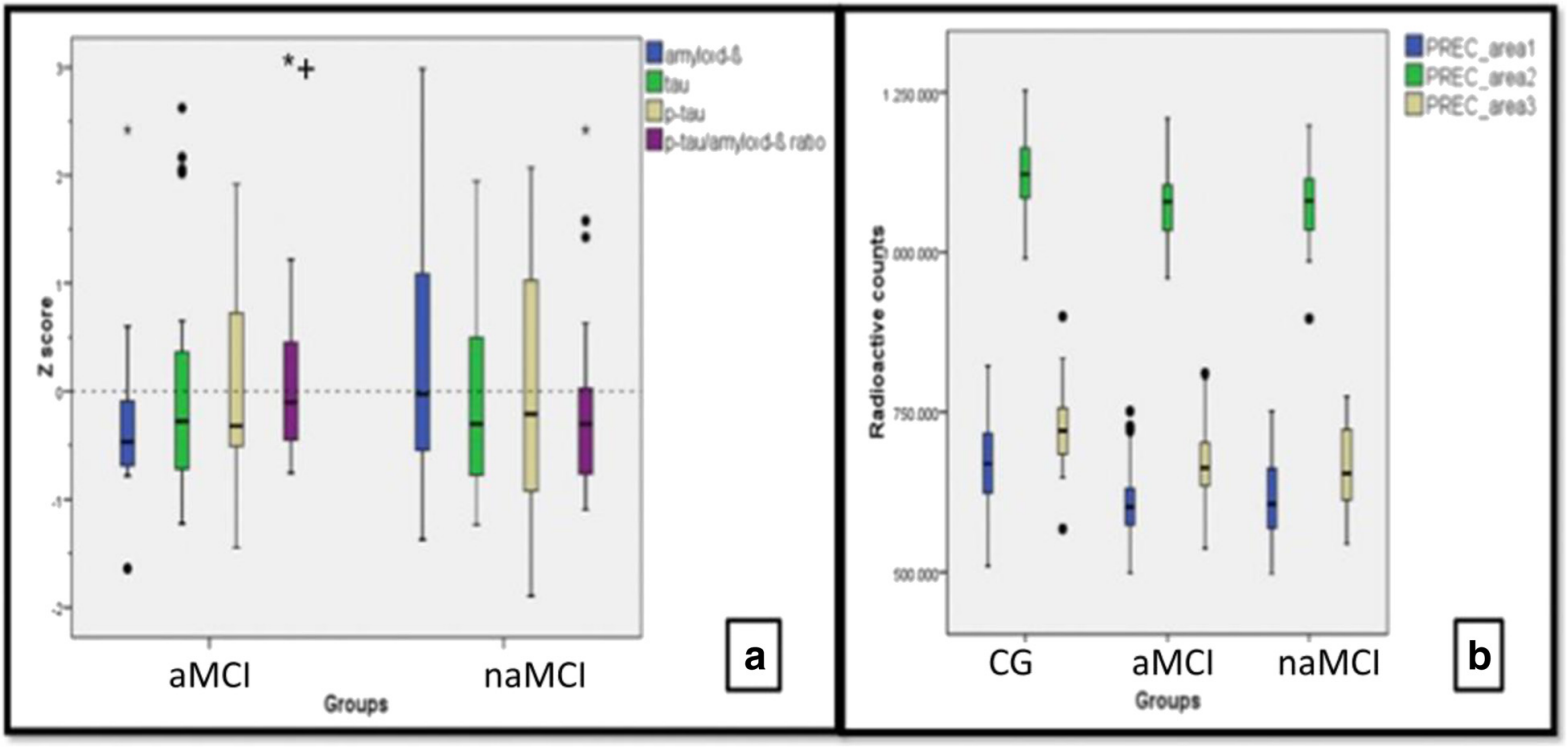
Box-plot graphics for CSF biomarkers and mean radioactive counts. **a** *Z* scores for CSF Aβ, tau, p-tau, and p-tau/Aβ ratio proteins in each group. **b** Mean radioactive counts in the three areas of the precuneus which presented rBGM differences between aMCI and CG (PREC_area 1 and 2) and naMCI and CG (PREC_area 3). ^•^1.5 times the interquartile range, *more than 1.5 times the interquartile range; ^+^value is actually 5.1 SD (outlier). *aMCI* amnestic mild cognitive impairment, *CG* control group, *naMCI* nonamnestic mild cognitive impairment, *p*-*tau* phosphorylated tau

In the subsample with available lumbar puncture (*n* = 54; aMCI, 28 patients; naMCI, 26 patients), CSF biomarker analysis depicted lower concentrations of CSF Aβ peptide in aMCI in relation to naMCI (M (SD) = 704 (248) and 918 (430), respectively; *p* = 0.04). No differences were found in tau, p-tau, and p-tau/Aβ protein ratio measures. To facilitate the visualization of the dispersion of CSF biomarkers values among aMCI and naMCI patients, Fig. 1a shows box-plot graphics of the *Z* scores (two groups combined) for CSF Aβ, tau, p-tau, and CSF Aβ/p-tau ratio.

Complete SPM8 data are presented in Table 2 and comprise the region of rBGM alterations in the ANOVA test and rBGM reductions in the post-hoc analysis, as well as its extension (number of voxels) and level of statistical significance. When compared with CG, naMCI exhibited statistically significant [^18^F]FDG-PET rBGM reductions in the following Broadmann areas (BAs): right middle frontal gyrus (BA8 and BA9, *p* <0.001; and BA10, *p* = 0.001), right inferior frontal gyrus (BA9, *p* <0.001), two contiguous areas in the precuneus (BA7, *p* = 0.001), and another area in the left superior occipital gyrus (BA19, *p* <0.001). The naMCI group exhibited reduced rBGM in right middle frontal gyrus (BA46, *p* <0.001) in relation to aMCI.

**Table 2:** rBGM comparison between aMCI, naMCI, and CG using SPM8^a^. Results showing areas of rBGM reduction

	*Z* score	*p* value	Cluster size (number of voxels)	Peak voxel coordinates (Talairach)		
aMCI × CG^b^						
Left middle temporal gyrus, BA39	3.51	<0.001	41	−46	−67	20
Left precuneus, BA31	3.17	<0.001	33	−4	−51	32
Left precuneus, BA7	3.33	<0.001	15	−2	−63	64
aMCI × naMCI^b^						
Left temporal lobe	3.62	<0.001	167	−44	−45	2
naMCI × control group^b^						
Right inferior frontal gyrus, BA9	3.80	<0.001	34	46	11	29
Right middle frontal gyrus, BA9	3.40	<0.001	51	34	34	28
Right middle frontal gyrus, BA8	3.32	<0.001	51	32	29	39
Right middle gyrus, BA10	3.24	0.001	11	38	45	16
Left superior occipital gyrus, BA19	3.42	<0.001	41	−42	−72	28
Left precuneus, BA7	3.20	0.001	32	0	−63	60
Left parietal lobe, BA7	3.16	0.001	32	−4	−62	70
naMCI × aMCI^b^						
Right middle frontal gyrus, BA46	3.33	<0.001	19	51	29	26

^a^Results at the peak voxel level (global analysis, analysis of variance and post-hoc nonpaired *t* test). SPM8 software from Wellcome Department of Cognitive Neurology (Functional Imaging Laboratory, London, UK)

^b^Uncorrected for multiple comparisons

*aMCI* amnestic mild cognitive impairment, *BA* Broadmann area, *CG* control group, *naMCI* nonamnestic mild cognitive impairment, *rBGM* regional brain glucose metabolism

**Fig. 2:**
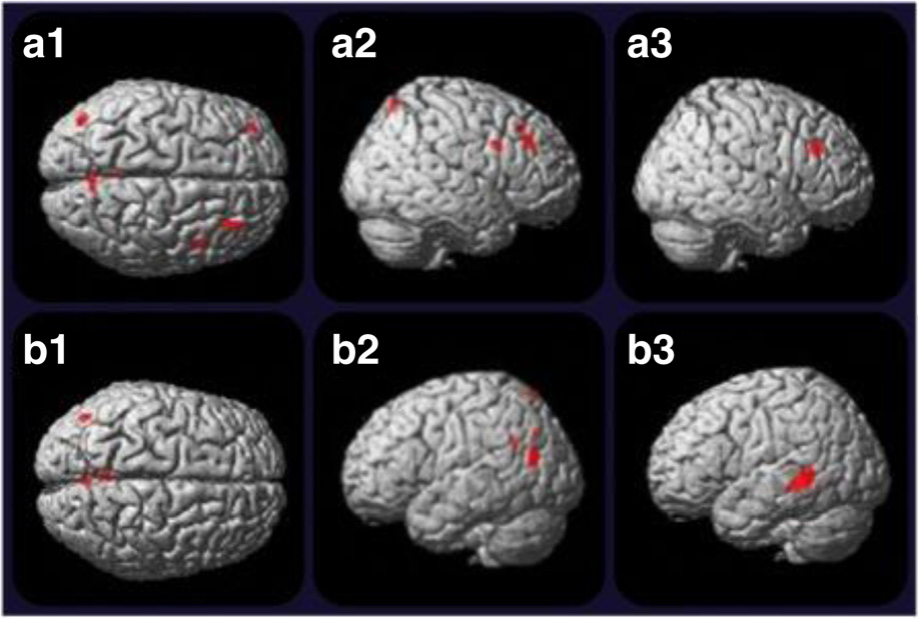
Illustrative anatomic localization of the peak voxels of rBGM reductions as measured with [^18^F]-FDG-PET. **a1**, **a2** naMCI rBGM reductions in relation to the CG, predominantly in right prefrontal areas but also in the precuneus and a left prefrontal area without statistic significance; **a3** naMCI rBGM reductions in relation to the aMCI group (right prefrontal area). **b1**, **b2** Bilateral metabolic reduction in the precuneus, parietal, and temporal cortex is seen in the aMCI group in comparison with normal older subjects; **b3** hypometabolism is also noted in the left temporal lobe in aMCI in relation to naMCI

The aMCI group showed decreased rBGM in relation to CG in the left precuneus (BA7 and BA31, *p* <0.001) and in the left middle temporal gyrus (BA39, *p* <0.001). When compared with naMCI, aMCI showed reduced rBGM in the left temporal lobe (*p* <0.001). Figure 2 shows an illustrative anatomic localization of the peak voxels of rBGM reductions as measured with [^18^F]-FDG-PET.

Results from the ANOVA indicated a significantly larger number of years of education for the control group relative to aMCI subjects (*p* = 0.01, CG >aMCI). The ANOVA also showed a statistical tendency for the CG to be slightly older than aMCI subjects (*p* = 0.06). Because these variables—especially education—could possibly influence the metabolic pattern in prodromal AD [32, 33], an analysis controlling for age and education was conducted; the results were not significantly changed after controlling for age and education.

SVC analysis of the temporoparietal association cortex of the aMCI and naMCI groups against the CG disclosed significant areas of rBGM reduction, after correction for multiple comparisons (family-wise error correction method (FWE)), in the left precuneus (BA31) in the aMCI group (*p*_FWE_ = 0.025) and in two contiguous areas in the left angular gyrus (BA39) in the naMCI group (*p*_FWE_ = 0.033 and 0.040).

The authors also designed ROIs to extract mean radioactive counts of the areas with statistically significant rBGM reduction in the precuneus of the three groups (as detailed in Materials and methods). Box-plot graphics were also generated in efforts to investigate the possible heterogeneity of neurodegeneration in these areas across all subjects. The results are shown in Fig. 1b.

## Discussion

Our results showed that naMCI patients presented a reduction of rBGM in the right prefrontal cortex relative to control patients, and in the right middle frontal gyrus relative to aMCI patients. Both MCI groups presented decreased rBGM with similar localization of decline, in the precuneus, in relation to the CG. Additionally, the aMCI group had significantly lower levels of CSF Aβ peptide, indicating increased deposition in the brain, and decreased left temporal lobe metabolism relative to the naMCI group.

Reduction of rBGM in the precuneus was seen in both the aMCI and naMCI groups relative to the CG. This area is believed to be an important component of the default mode network, a network that is structurally and functionally affected in normal aging and more prominently, and very early, in AD [34–36]. The precuneus and posterior cingulate cortex seem to be particularly vulnerable to Aβ deposition and metabolic dysfunction [36, 37]. Lower rBGM and cerebral blood flow in this region are commonly associated with a faster rate of progression from MCI to AD [28, 30, 31, 38].

These results indicate that both the aMCI and naMCI groups have significant rBGM reductions in the posteromedial parietal cortex relative to the CG. These data are in line with Clerici et al. [9], who found that subjects with single-domain aMCI and naMCI have significant medial parietal cortex (posterior cingulate cortex) hypometabolism in relation to normal older subjects. The authors also reported additional metabolic reductions in the medial temporal lobe of aMCI in relation to naMCI, which was not replicated in the results outlined in the present article. However, our results do indicate that aMCI subjects still had a reduction of rBGM in the left temporal lobe in comparison to naMCI (Table 2).

Our findings—that there is an rBGM reduction in the right prefrontal areas in the naMCI group in relation to the CG, and to a lesser extent in relation to the aMCI group—suggest there is some relation with an rBGM reduction in the right prefrontal areas and the impairments of executive function and attention, but not memory deficits, presented by naMCI patients. The dorsolateral prefrontal cortex is classically associated with executive functions and has strong anatomic and functional connections to attentional and executive brain networks [39, 40]. These findings are in agreement with previous studies that revealed blood flow and volumetric reductions in frontal lobes of patients with naMCI [10, 11].

A volumetric reduction in the left dorsolateral prefrontal cortex was shown in single-domain dysexecutive MCI relative to the control group [11]. Nobili et al. [10] reported blood flow reductions in the right frontal cortex of naMCI patients as compared with patients with subjective memory complaints. However, methodological differences such as a high rate of depression and WMH in the naMCI group, use of SPECT instead of PET, and the analysis of preselected ROIs disallow an easy comparison between the results presented in the present study and those published previously. In the former study [10], vascular disease or depression may have accounted in part for the frontal hypoperfusion. Small white matter and subcortical vascular lesions correlate negatively with dorsolateral prefrontal cortex activity, and thus could be responsible for executive and attentional dysfunctions [41, 42]. As described, any subjects with clinically diagnosed depression, depression that restricted normal daily activity, or cerebral infarcts were excluded. Notably, we found no differences in cerebrovascular risk factors and WMH intensity on MRI across the groups. Thus, even though MRI may miss cortical microinfarcts in some cases, vascular disease is unlikely to be the main cause of the frontal hypometabolism in the naMCI group [43].

aMCI subjects had lower levels of Aβ peptide as compared with the naMCI subjects (Table 1). A meta-analysis of the relationship between brain amyloid biomarkers in normal older adults suggested a closer relationship between brain amyloid with memory function than with other cognitive functions [44]. A similar correlation has been shown in aMCI but not naMCI patients [45]. The fact that lower Aβ levels in the CSF are thus linked to a greater deposition of amyloid plaques in the brain possibly indicates that the aMCI subgroup is more similar to a pathological state of AD than the naMCI group [46, 47]. This relationship further suggests that aMCI patients are more likely than naMCI patients to be diagnosed with AD in the future [48]. In line with our finding, lower cortical Aβ deposition measured with [^11^C]PIB-PET was also found in naMCI in comparison with aMCI [49].

There was no difference in tau and p-tau proteins levels between the aMCI and naMCI groups. The tau and p-tau proteins are associated with neurofibrillary tangles, which are more associated with synaptic dysfunction, brain atrophy, and cognitive and functional impairment than Aβ [8, 46, 47]. Together with brain atrophy and [^18^F]FDG-PET hypometabolism, tau and p-tau are classified as biomarkers of neural injury or degeneration [8]. The CSF tau and p-tau results are in partial agreement with the [^18^F]FDG-PET findings, showing similar precuneus hypometabolism in both MCI groups, thereby potentially indicating that the two MCI groups are not in different stages of neural degeneration. Interestingly, a recent study found cognitively normal subjects with positive biomarkers of neurodegeneration and no Aβ accumulation [50]. Those same subjects, however, later developed Aβ deposits [50], indicating that the trajectories of Aβ accumulation and injury biomarkers may be independent from one another [51]. These findings may help explain the results in the biomarker profile of the present study’s naMCI group, which showed similar neuronal injury biomarkers in both MCI groups but less brain Aβ deposition in naMCI patients.

The additional frontal hypometabolism in naMCI, however, remains an open question. Cerami et al. [52] recently reported a follow-up cohort study of MCI accessed with [^18^F]FDG-PET at diagnosis, including eight naMCI patients. In Cerami et al.’s study, two patients with naMCI and frontal hypometabolism later developed frontotemporal lobar degeneration (FTLD), implying that FTLD remains a possible explanation for the development of frontal hypometabolism in naMCI patients. The authors of the current study believe this explanation to be unlikely, however, primarily because none of our participants presented with behavioral changes, which are a major clinical feature in FTLD and were an exclusionary criterion in our study.

Executive dysfunction and impaired attention are both unspecific findings that are related to the prefrontal cortex [39, 40, 53], and may be present in several etiologies without concomitant neurodegeneration, such as in psychiatric diseases [53]. The hypometabolism seen in the naMCI group may therefore be due to a “functional network dysregulation” of the attentional and executive networks, although with still unknown origin.

Clinical follow-up and reappraisal of biomarkers for the naMCI group is crucial in determining the outcome and probable etiological diagnosis of naMCI patients. The naMCI group could be composed of patients with different etiological entities, such as aging-associated executive and processing speed dysfunctions [37], prodromic presentation of non-AD neurodegenerative diseases [52], atypical presentations of AD (e.g., frontal executive AD) [54], nondegenerative cognitive impairment (e.g., vascular disease), and even preamnestic forms of MCI owing to AD. Follow-up is fundamental in drawing any conclusions about this issue. Taking into account the possibility of non-Aβ degeneration [50, 51] and the fact that executive function is an unspecific finding, naMCI is probably a different type of degeneration than aMCI, not necessarily a milder one. This distinction can only be clarified with a long-term follow-up.

Our study has some limitations. First, because naMCI probably represents a heterogeneous group, additional studies with larger patient samples are needed. Second, additional investigations of naMCI samples should be performed using other imaging biomarkers modalities, such as volumetric analysis with MRI, and other variables, such as genetic analysis, measures of cognitive reserve, lifestyle, and so forth. Third, [^18^F]FDG-PET imaging was acquired in the resting state. For that reason, discussions relating rBGM to neuropsychological functions (i.e., frontal hypometabolism and executive function) should be considered only speculative; more investigation is warranted before these observations can be confirmed. Finally, our patient population was a convenience sample from the community; future studies would benefit from incorporating a variety of different populations (e.g., subjects from a tertiary memory clinic).

## Conclusions

The naMCI and aMCI groups presented some similarities in their biomarker profile. Both groups presented the same levels of tau and p-tau proteins in the CSF and a reduction of rBGM in parietal areas (mainly the precuneus) relative to controls. However, some important differences were noted between the two groups: aMCI patients had lower levels of Aβ peptide in the CSF and left temporal hypometabolism and increased right prefrontal cortex rBGM relative to naMCI patients. Ultimately, these results illustrate that aMCI patients had a biomarker profile more similar to MCI owing to AD, while naMCI patients illustrate a different—and varied—pattern of degeneration.

## Abbreviations

[^18^F]FDG-PET: Positron emission tomography with 18F-fluordeoxyglucose
AD: Alzheimer’s disease
aMCI: Amnestic mild cognitive impairment
Aβ: Amyloid beta
ANOVA: Analysis of variance
BA: Broadmann area
CG: Control group
CSF: Cerebrospinal fluid
FTLD: Frontotemporal lobar degeneration
FWE: Family-wise error correction method
GDS: Geriatric Depression Scale
M: Mean
MCI: Mild cognitive impairment
MMSE: Mini-Mental State Examination
MRI: Magnetic resonance imaging
naMCI: Nonamnestic mild cognitive impairment
p-tau: Phosphorylated tau
rBGM: Regional brain glucose metabolism
ROI: Region of interest
SD: Standard deviation
SPM: statistical parametric mapping
SVC: Small volume correction
WMH: White matter hyperintensities
